# A Rapid Nanofocusing Method for a Deep-Sea Gene Sequencing Microscope Based on Critical Illumination

**DOI:** 10.3390/s24155010

**Published:** 2024-08-02

**Authors:** Ming Gao, Fengfeng Shu, Wenchao Zhou, Huan Li, Yihui Wu, Yue Wang, Shixun Zhao, Zihan Song

**Affiliations:** 1 Changchun Institute of Optics, Fine Mechanics and Physics, Chinese Academy of Sciences, Changchun 130033, China; 2 University of Chinese Academy of Sciences, Beijing 100049, China; 3 State Key Laboratory of Applied Optics, Changchun 130033, China; 4 Key Laboratory of Optical System Advanced Manufacturing Technology, Chinese Academy of Sciences, Changchun 130033, China

**Keywords:** deep sea, critical illumination, auto-focus, edge gradient, gene sequencing

## Abstract

In the deep-sea environment, the volume available for an in-situ gene sequencer is severely limited. In addition, optical imaging systems are subject to real-time, large-scale defocusing problems caused by ambient temperature fluctuations and vibrational perturbations. To address these challenges, we propose an edge detection algorithm for defocused images based on grayscale gradients and establish a defocus state detection model with nanometer resolution capabilities by relying on the inherent critical illumination light field. The model has been applied to a prototype deep-sea gene sequencing microscope with a 20× objective. It has demonstrated the ability to focus within a dynamic range of ±40 μm with an accuracy of 200 nm by a single iteration within 160 ms. By increasing the number of iterations and exposures, the focusing accuracy can be refined to 78 nm within a dynamic range of ±100 μm within 1.2 s. Notably, unlike conventional photoelectric hill-climbing, this method requires no additional hardware and meets the wide dynamic range, speed, and high-accuracy autofocusing requirements of deep-sea gene sequencing in a compact form factor.

## 1. Introduction

Approximately three-quarters of Earth’s surface is covered by oceans, with half being deep-sea regions where the depth surpasses 1000 m [1]. These zones are characterized by the absence of light and the presence of water temperatures that are close to freezing. However, they also exhibit the presence of hydrothermal vents that erupt at temperatures that exceed 400 °C [2,3]. The deep-sea environment is characterized by extreme pressure, with the Mariana Trench representing a depth of 1100 atmospheres. Salinity levels are also elevated in this milieu [4]. Microbes that thrive in these extreme conditions, known as extremophiles, possess genetic sequences that are not yet fully understood by scientists. These sequences enable the microbes to produce enzymes resilient to diverse temperatures, salinity levels, pH, and pressures [5,6]. The discovery of these genetic sequences is expected to have a significant impact on various sectors, including agriculture, pharmaceutical manufacturing, and the field of bioengineering [7,8].

Despite the significant progress that has been made in the field of deep-sea microbial genetics, the majority of analyses are conducted on land following the extraction of samples from the deep sea [9,10]. The transition in the environment has the unintended consequence of introducing deviation into the results [11,12]. This is particularly problematic in the context of deep-sea genetic sequencing, where the lack of specialized equipment represents a significant challenge [13,14]. In contrast to laboratory-based sequencers, which operate in stable temperatures and humidity, those deployed in the deep sea must endure isolated, extremely harsh conditions over long periods. These instruments are limited in space by the compression of the cabin and must withstand severe shock during descent. They operate fully automatically on the sea floor for months or even a year. The temperature inside the cabin is in the range of 0 °C to 20 °C. During that time, they cannot be maintained [15,16,17]. There is a critical need to develop autofocusing mechanisms that are highly integrated, resilient, and possess a wide detection range to ensure the successful operation of in situ deep-sea gene sequencing equipment.

Conventional sequencers employ photoelectric sense systems to localize the focal plane [18,19,20,21]. Zhang proposes two laboratory autofocusing methods based on multi-position laser differential confocal and laser-based arrayed spots, respectively. The former has a focusing accuracy of 1/10 of the depth of focus, and the latter has a dynamic range of ±100 μm, but not both [22,23]. However, these approaches are often structurally complex and offer inadequate dynamic ranges. In such systems, light emitted from a dedicated source is reflected off the imaging surface and detected, enabling the determination of the out-of-focus surface’s position. This information is then used by an actuator to correct any defocus. While these techniques share an optical path with the imaging system, either entirely or in part, substantial temperature shifts and severe impacts may cause the systems to lose cofocus. It would be very challenging to recalibrate in a deep-sea environment [24,25,26,27,28].

This study introduces a focus detection method that identifies the edges of the critical illumination field under various defocus conditions and is seamlessly integrated with the gene sequencing imaging system. Differing from traditional, edge detection approaches, our method pins down the gradient extremities to discern the defocus by measuring the position shift between the field’s opposing edges. This difference serves as the metric for evaluating defocus. The system employs its built-in detector to capture the differences in the illumination field’s size across varying degrees of defocus. These differences are then used to generate a standard evaluation curve, which correlates the defocus amount with the metric. This curve is used to determine the autofocus mechanism precisely. Experiments on the deep-sea genetic sequencing autofocus microscope prototype substantiated the approach’s efficacy. The results indicate that within a dynamic range of ±40 μm, the accuracy of detection of a single defocused image can reach 200 nm. Moreover, the accuracy can be raised to 78 nm by accumulating multiple images through continuous sampling. Additional iterations can broaden the dynamic range beyond ±100 μm after two cycles. This robust method, which is impervious to perturbations such as laser energy variation and displacement, represents a viable autofocusing solution for deep-sea, in situ genetic sequencing.

## 2. Methods

The optical apparatus in the epi-illumination fluorescence microscope is designed to operate using two techniques: Köhler illumination and critical illumination [29,30,31]. In the field of genetic sequencing, critical illumination is preferred due to its superior ability to modulate the shape, depth, intensity, consistency, and homogeneity of the excitation light field [32,33,34]. Genetic sequencing scanners employ square critical illumination light fields, thereby enhancing the utilization factor of the sequencing chips. Concurrently, excitation light reflected off sequencing chips is typically regarded as noise and is consequently filtered out. In this study, we exploit these extraneous light fields, utilizing them as a focus detecting optical signal within the system’s autofocus feature.

**Figure 1:**
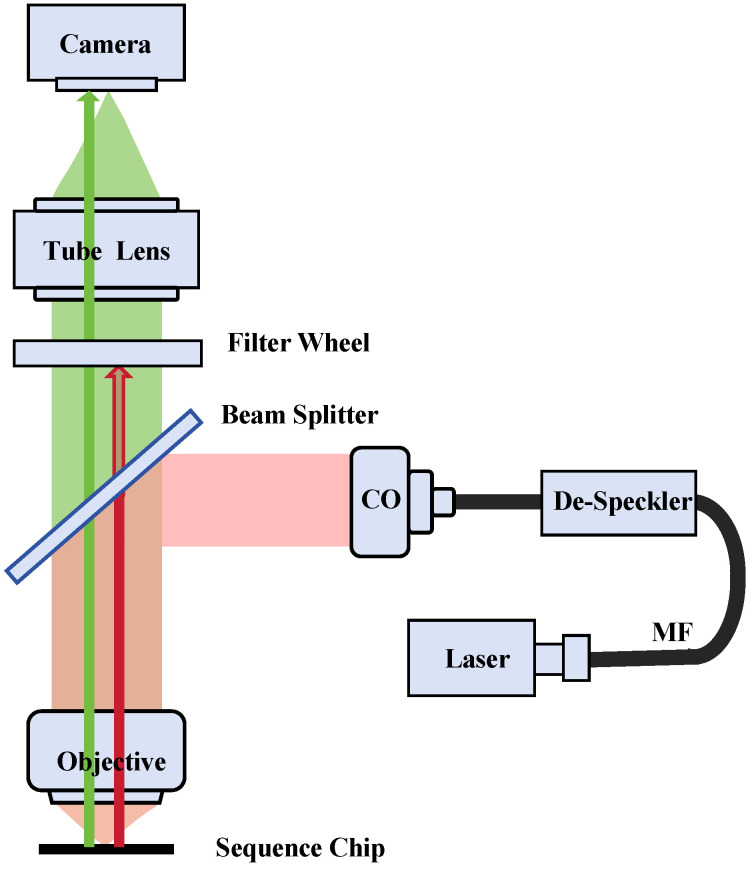
Critical illumination fluorescence microscopy imaging system. CO is a collimator. MF is a multimode optical fiber with a square output section. De-Speckler is a speckle-beam homogenizer.

Figure 1 illustrates the optical configuration of a multichannel fluorescence microscope optimized for genetic sequencing. The system comprises an infinity-corrected objective tube lens, and a camera, collectively establishing an imaging mechanism. The laser illumination part of the prototype imaging system consists of a collimating lens, a laser, a square multimode fiber, and a diffuser. The diffuser facilitates beam homogenization. Laser illumination, after transmission through the multimode fiber and collimation into a parallel beam, is focused by the microscope objective onto the genetic sequencing chip at the focal plane. The gene sequencing chip consists of a silicon substrate and a glass cover, with channels spaced 170 μm apart to facilitate the growth of DNA clusters on the silicon surface. The silicon’s surface serves as a reflective mirror, redirecting the excitation light back into the objective lens. To discern fluorescence signals within specific spectral ranges while excluding extraneous light from reflected laser beams, the microscope’s detection system employs a filter wheel. In the empty channel, this wheel permits reflected laser light to strike the detector’s surface directly, generating a focused detection signal.

**Figure 2:**
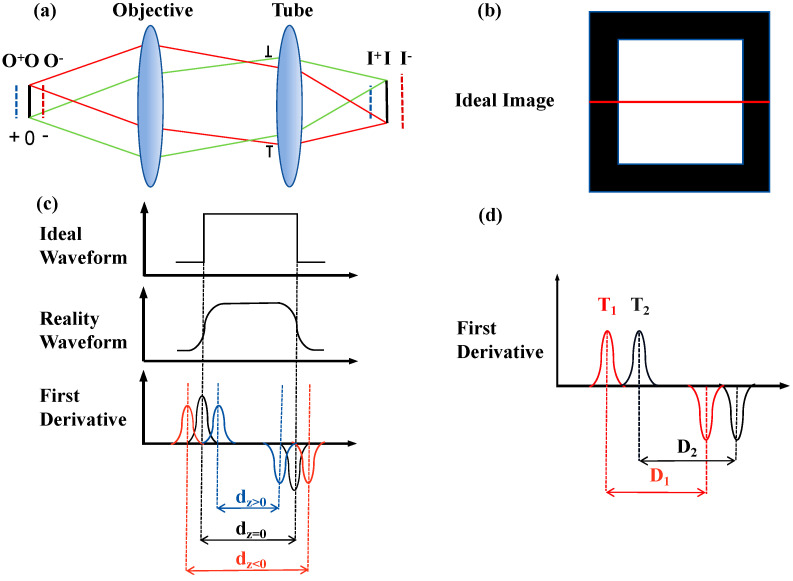
The principle of critical illumination focuses on feedback. (**a**) The optical path for the infinite-range microscope. The red and green lines represent the propagation path of light rays at the edge of the field of view. (**b**) Simulation of square laser illumination output end-face field distribution. (**c**) Intensity and gradient simulation distribution at the line position in (**b**). (**d**) The gradient distributions T_1_, T_2_ and the positional difference of the gradient extrema D_1_, D_2_ before and after the displacement of the light field.

Figure 2a depicts the optical imaging pathway of the genetic sequencing microscope, which includes both an objective lens and a tube lens. The critical illumination system directs the light source onto the focal plane of the objective. In the absence of a cutoff filter, the conjugate image materializes on the focal plane of the eyepiece, as shown in Figure 2b. The diagram above Figure 2c displays the one-dimensional, grayscale intensity profile of the conjugate image’s square critical illumination light field outlined in Figure 2b. As the distance of the object alters, adjusting the image distance is necessary to preserve optimal imaging. While sharp images are obtainable despite alterations in the object and image distance, concurrent changes in magnification are inevitable. The amount of out-of-focus z is defined as 0 at the focal plane of the lens, with negative values closer to the lens and positive values further from the lens. In real-world applications, due to the invariable image distance, defocus arises. Figure 2c is a diagram of the location of the lines shown in Figure 2b. Figure 2c portrays the actual one-dimensional grayscale profile of the defocused light field, and the lower diagram depicts its first-order derivative or gradient profile. Defocusing results in blurring of the boundaries of the light field, yet the gradient extremes remain. The separation between these extremes depends on both the magnification and the state of defocus of the system. As indicated in Figure 2c, the optimal focus is attained when the separation d_z_ is zero. Defocusing alters the magnitude and position of the edge gradients due to edge blurring and changes in magnification.

In light of these observations, we introduce an innovative autofocus approach for genetic sequencing, leveraging the critical illumination light field as an optical feedback signal. This strategy not only streamlines the complexity of the system and shrinks its spatial footprint but is also unaffected by displacement of the light field. Figure 2d depicts the concept behind this interference immunity. The deployment and recovery process of deep-sea gene sequencers is accompanied by strong shocks that cause changes in the position of the light field. Since the excitation light for the sequencer comes from compact, rigid laser emission surfaces such as optical fibers or light homogenizing rods, the polar position difference D_1_ is equal to D_2_.

## 3. Results

### 3.1. Simulation Analysis

The calculation of image gradients represents a pivotal methodology for the identification of edge information in visual data. This process quantifies the change in image sizes, whereby regions exhibiting edges exhibit elevated gradient magnitudes due to marked variations in intensity. Edge classes are predominantly comprised of step, ridge, slope, and pulse variations [35,36]. The different categories are rooted in the speed of intensity change. The edge of the critically illuminated field is the ridge shape with the fastest intensity change. Figure 2c displays both theoretical and practical profiles of ridge-type edge fluctuation, in addition to the gradient estimation curve derived from first-order derivative edge detection operators. The process of gradient computation involves the calculation of derivatives. Nevertheless, given the discrete nature of image matrices, such changes are estimated via differential approximations. The approximate values of gradients—essentially estimated derivatives—are determined by gauging the variation in pixel intensity [37,38]. For any given pixel with luminance $g\left(x,y\right)$, its gradient magnitude can be expressed as follows:

(1) $$f=\sqrt{G_{x}{\left(x,y\right)}^{2}+Gy{\left(x,y\right)}^{2}}$$

Gradient (difference) in the x-direction:

(2) $$G_{x}=g\left(x+1,y\right)-g\left(x,y\right)$$

**Figure 3:**
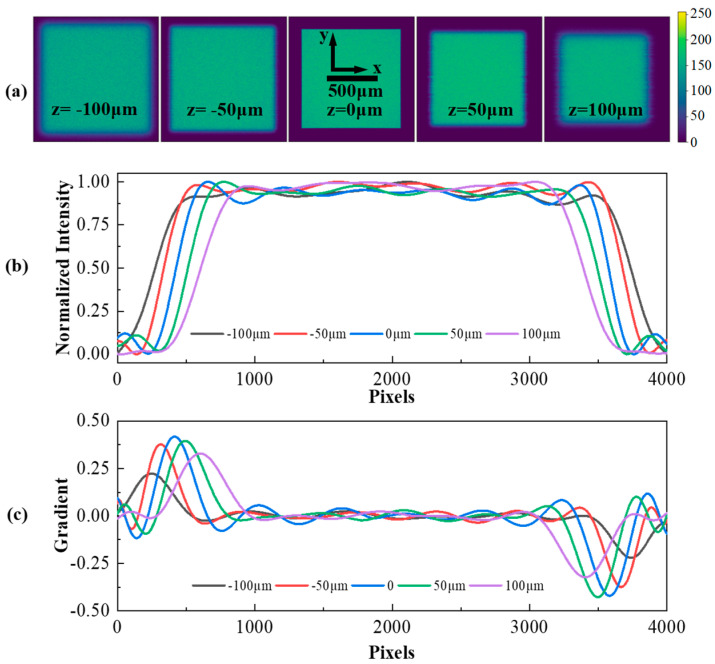
Simulation of the defocused critical illumination light field. (**a**) Conjugate images of the excitation field at five defocused positions. (**b**) One−dimensional intensity curves in the x-direction of the conjugate images of the excitation field at five defocused positions. (**c**) One−dimensional gradient curves in the x−cut direction of the conjugate images of the excitation field at five defocused positions.

To validate our analysis, critical illumination microscopy was performed using ZEMAX2013 to obtain conjugate images of defocused critical illumination light fields at different defocus positions, as shown in Figure 3a. The simulation was performed using a 30x infinity microscope objective model with f = 8 mm, a laser collimator with f = 12 mm, and an air-spaced achromatic doublet with f = 200 mm as a converging tube lens. The light source size is 1 × 1 mm; the detector size is set to 20 × 20 mm with 4000 × 4000 pixels. The intensity distribution along the *x*-axis at the same location, as shown in Figure 3a, is presented in Figure 3b. Figure 3c illustrates the application of a first-order derivative edge operator to analyze the gradient in the simulated conjugate images for differing defocus states. The results indicate a notable decline in the magnitude of edge gradient extremes (maxima and minima) and a corresponding shift in their positions as defocus intensity is increased. A robust correlation was discerned between the magnitude of edge gradients, their positions, and defocus levels.

**Figure 4:**
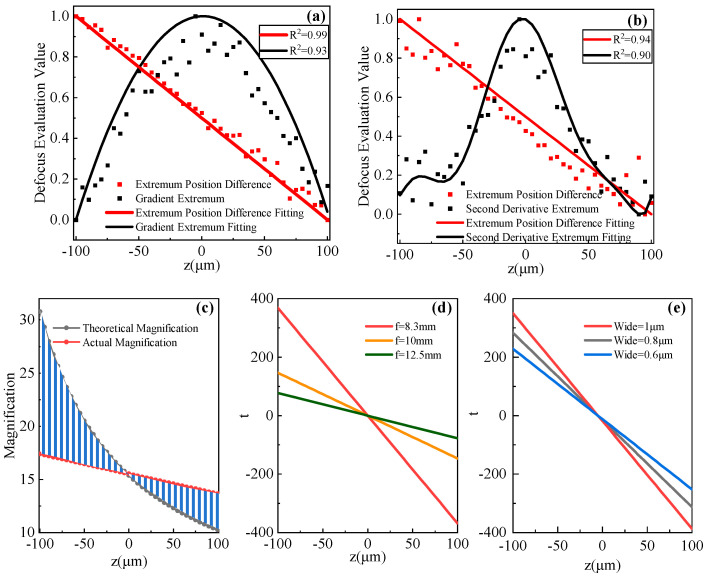
Simulation of standard evaluation curves for defocus distances. (**a**) The black line is the normalized standard defocus distance evaluation curve, plotted using the gradient extremes as the defocus evaluation value. The red line is the normalized standard defocus distance evaluation curve, plotted using the gradient extremum position difference as the defocus evaluation value. (**b**) The black line is the normalized standard defocus distance evaluation curve, plotted using the second derivative extremum as the defocus evaluation value. The red line is the normalized standard defocus distance evaluation curve, plotted using the second derivative extremum position difference as the defocus evaluation value. (**c**) The theoretical magnification of the system under defocusing and the actual magnification obtained from edge recognition. (**d**) Standard evaluation curves for different objective lens focal lengths. (**e**) Standard evaluation curves for different illumination widths.

This paper describes an optoelectronic autofocus method. This type obtains the defocus evaluation value by processing and calculating the detected optical or electrical signals to obtain the defocus evaluation value. The method in this paper detects the one-dimensional energy distribution of the optical field under critical illumination conditions and calculates the positional difference between the maximum and minimum values of the gradient of the energy distribution as the differential defocus evaluation value. This value is essentially a distance or width metric. Later we will use “t” to represent the defocus evaluation value, for example, the vertical coordinate in Figure 4d. Before the first autofocus is performed, a series of equally spaced statistics corresponding to a known defocus evaluation value is required, and a standard defocus evaluation curve is constructed by fitting these data. This curve shows the direct relationship between the defocus distance and the defocus evaluation value. Figure 4a shows two normalized standard defocus evaluation curves. In the autofocus process, the current defocus evaluation value is first obtained by detection and calculation, and then the standard evaluation curve is used to determine the degree of defocus, which is finally compensated by the actuator.

Standard evaluation curves were individually plotted to demonstrate these observed patterns, as shown in Figure 4a. The evaluation curve correlating with gradient extremes displays a near-quadratic relationship, peaking around the focal plane. This results in a drastic reduction in focus sensitivity within its vicinity, which impedes the ability to discern defocus direction from a solitary assessment and adversely affects the accuracy and efficiency of autofocus. The focus sensitivity is quantified as the slope of the evaluation curve. Conversely, the evaluation curve associated with positional differences of gradient peaks closely resembles a linear relationship, suggesting consistent focus sensitivity across the entire dynamic range. This enables enhanced one-shot autofocus accuracy, as the linearity of the curve allows for a more accurate estimation of focus. We also performed a simulation analysis using grayscale, second-order derivative poles and their positions. The higher order derivative analysis exhibits higher sensitivity, as evidenced by the faster rate of change of the second-order derivative extremes than the first-order derivative extremes in Figure 4b. However, the dynamic range is significantly reduced and more susceptible to noise, resulting in a significant increase in bias. The second-order grayscale derivatives at the edges of both sides of the optical field also show extremes with the same position difference as the first-order derivatives. It can be seen that the sensitivity of the focusing method based on extreme position differences is not improved by the use of higher-order derivative analysis but can lead to a larger deviation.

In optimal imaging conditions, variations in object distance prompt corresponding adjustments in image distance, allowing for an exact calculation of system magnification via Newton’s formula. However, in cases where image distance does not vary with object distance, system magnification calculation fails when the system is defocused. In such instances, the edge is usually the location of the gradient extremes. Consequently, by measuring the inter-edge distance to determine the image size and comparing it with the object size, we can derive the system magnification at that particular focus. Figure 4c illustrates the relationship between magnification under such circumstances and the deviation from the intended focal plane. It demonstrates an increase in the discrepancy from ideal magnification with enhanced defocus distance and a consequent diminished change in magnification tending towards linearity.

The slope of the standard evaluation curve is indicative of focus sensitivity. We conducted a thorough analysis of the various elements that influence this sensitivity. The positional disparity of the peak gradient value at zero defocus is designated as d_0_, and the defocus evaluation metric t represents the difference between the positional deviation d of the peak gradient value at defocus z and d_0_. We created standard evaluation curves of t against defocus z. As illustrated in Figure 4d, these curves are derived from sampling at 5 μm intervals within a ±100 μm defocus range and utilizing objectives with various focal lengths. It indicates that objectives with shorter focal lengths exhibit a higher absolute value of the slope in the standard evaluation function. This is attributable to their larger magnification changes in response to equivalent object distance alterations, thereby enhancing focus sensitivity.

The grayscale data captured by the detector is discrete, which means that the smallest discernible change in the position of gradient extrema is one pixel. This one-pixel variation corresponds to the smallest detectable defocus amount, which defines the system’s detection limit (peak sensitivity). To enhance sensitivity, it is possible to reduce the pixel size and increase the quantity, or alternatively, to expand the size of the critical illumination excitation light field in order to cover more pixels under the same magnification change conditions, as illustrated in Figure 4e.

### 3.2. Accuracy Test

**Figure 5:**
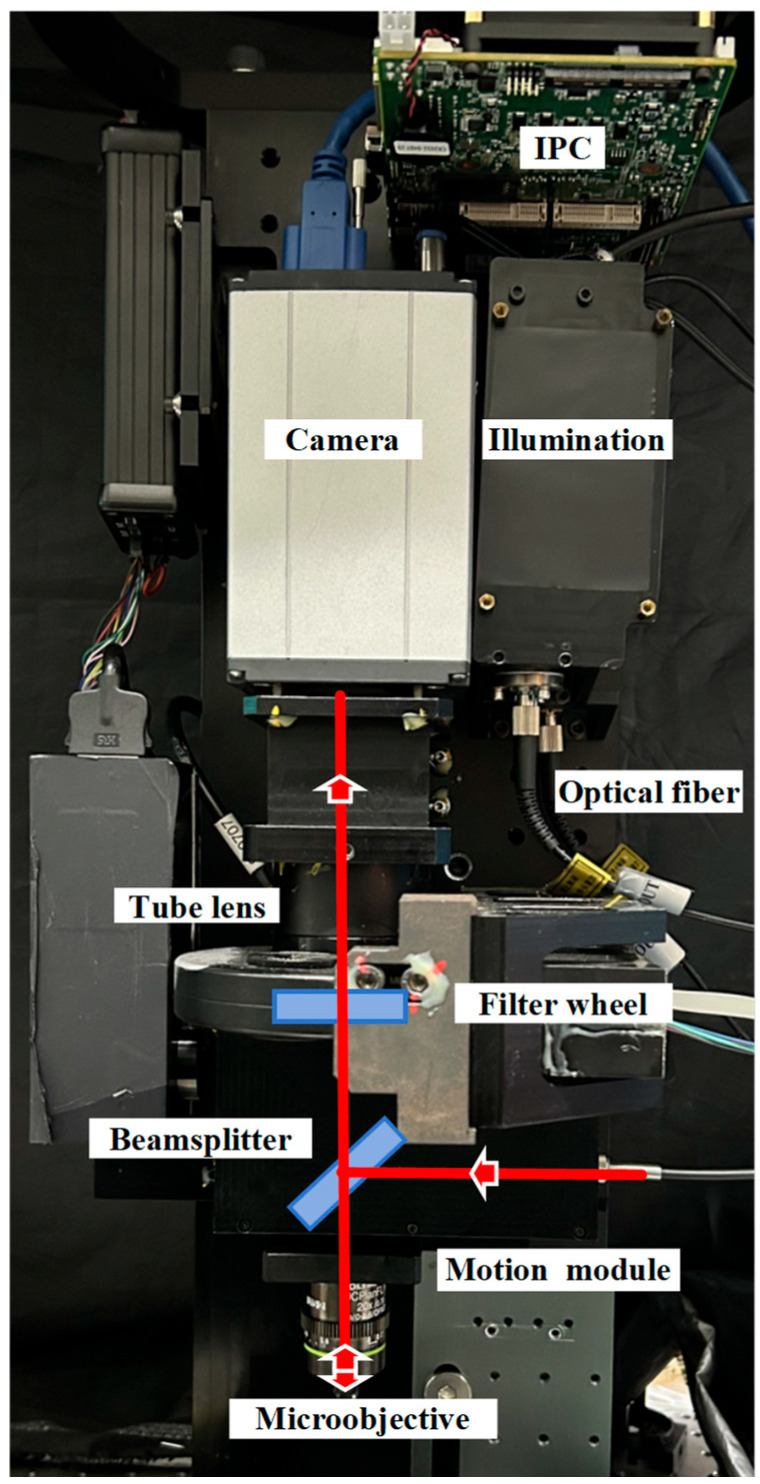
Prototype of a multichannel fluorescence microscope for gene sequencing. IPC: Industrial Personal Computer. Camera: Andor Zyla 4.2, Andor Technology, Belfast, UK. Illumination: semiconductor laser (Changchun New Industries, MDL-E-655, Changchun, China). Tube lens: Thorlabs TTL100-A, Thorlabs Inc., Newton, NJ, USA. Filter wheel: Thorlabs FW102C. Optical fiber: Changchun New Industries multimode optical fiber. Beamsplitter: Chroma ZT532/660rpc, MEETOPTICS, Barcelona, Spain. Microobjective: Olympus UCPLFLN20X (Olympus, Tokyo, Japan) with a numerical aperture of 0.7 and a magnification of 20×. Motion module: A one-dimensional linear displacement mechanism (WDI, ZAA-STD) was selected to provide a travel range of 10 cm with a minimum step displacement of 78 nm. The laser enters the color from right to left in the direction of the arrow and then shines downward. Reflected light propagates from bottom to top, with some continuing to propagate upward through the dichroic film.

The prototype of the multichannel fluorescence microscope for gene sequencing constructed according to Figure 1 is shown in Figure 5. The functional area of a gene sequencing chip is much larger than the imaging field of view, so a two-dimensional moving platform must be used to scan all the DNA clusters. After moving to each new location, the system first determines the focal plane. Once the illumination laser is turned on, the light is transmitted through an optical fiber to a collimator and into the microscope, where a dichroic beam splitter then reflects the laser light into the objective lens. The collimator and objective form a critical illumination system that projects the light field from the square fiber port onto the chip surface. The laser light field is reflected from the chip surface, and the objective collects this light and directs it into the system. A portion of the intense laser light passes through a dichroic beam splitter and is imaged onto the target surface of the detector. The system then collects an image of the light field and extracts the difference in position of the gradient peaks of the image to generate a standardized evaluation curve and calculate the current amount of defocus, which is then compensated by a *z*-axis displacement actuator. The focusing process is fully automated with the detector taking 40 ms to acquire an image, the actuator compensating the defocus amount in less than 100 ms, and the control program and algorithm calculating the amount in about 20 ms. The total time for a single focusing iteration is less than 160 ms. After focusing, the system turns the illumination laser back on and rotates the filter wheel to obtain DNA cluster site images in four different spectral bands. The system turns the illumination laser back on and rotates the filter wheel to obtain images of the DNA clusters in four different spectral bands.

**Figure 6:**
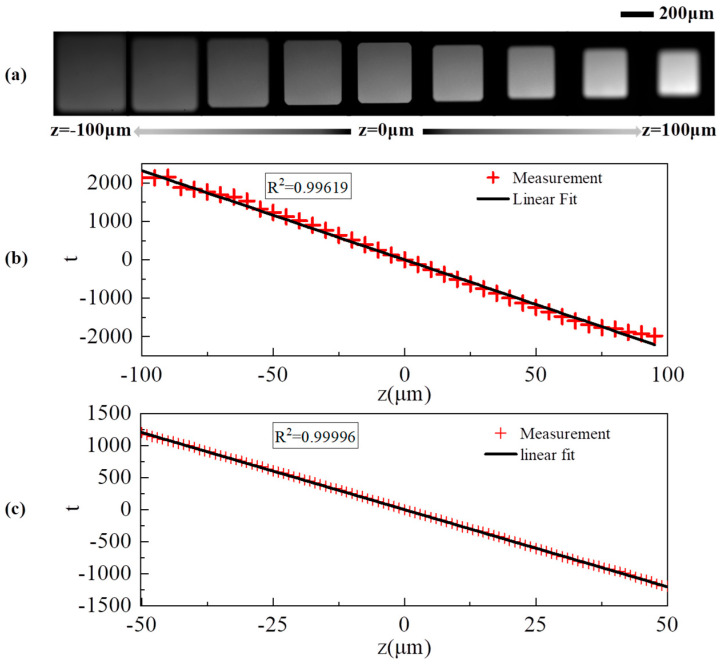
Extraction of standard evaluation curves for defocusing amounts. (**a**) Image of the excitation field of an equidistant defocused volume captured by the detector. (**b**) Standard evaluation curve of defocusing amount plotted at 5 μm intervals. (**c**) Standard evaluation curve of defocusing amount plotted at 1 μm intervals.

In our experiments, we stabilized the sequencing chip and modulated the objective to acquire conjugate images of the excitation field across various defocus stages. Images were captured at 1 μm intervals within a defocus range of ±100 μm, resulting in the acquisition of 200 images of the defocused excitation field. As illustrated in Figure 6a, images exhibiting defocus discrepancies of 25 μm were selected. It can be observed that the excitation field’s dimensions undergo a progressive reduction as transitions from negative to positive defocus values occur. Our previous simulation studies have indicated that reducing the pixel size or enlarging the excitation field’s area increases the number of pixels reacting to magnification changes, thereby enhancing sensitivity. To this end, we applied interpolation to the acquired defocused images. Figure 6b displays 40 images selected at 5 μm intervals from the original 200, which were employed in fitting a standard curve. This yielded an R^2^ value of 0.99619.

The experimental data exhibited exceptional linearity and minimal variance within a ±50 μm defocus range. Based on this, 100 images were selected at 1 μm intervals within a ±50 μm range to obtain the standard evaluation function for the defocus detection system, as shown in Figure 6c. The fitting of the standard evaluation function yielded an R^2^ value of 0.99996 and a slope (k) of −24.103/μm. The theoretical sensitivity of the defocus detection system is 1/k, which is approximately 0.041 μm.

**Figure 7:**
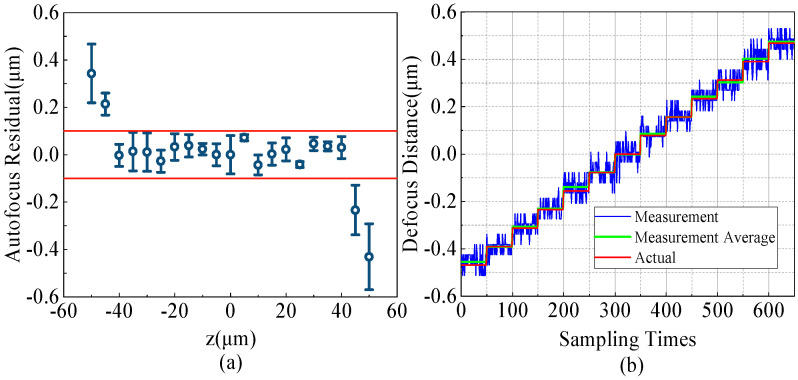
Focus accuracy test experiment. (**a**) Capture single images under different defocus conditions and calculate the amount of defocus, then determine the deviation from the theoretical value. The red line represents deviations of 0.1 μm and −0.1 μm. (**b**) Step out of focus 78 nm multiple times during continuous detection and record the amount of out−of−focus in real time.

Simulation analysis and Figure 6c indicate that the focus evaluation function exhibits monotonicity and a high degree of linearity. To evaluate the system’s single iteration focusing performance, a defocus quantification experiment was conducted with increments of 5 μm across a 50 μm range, involving 11 predetermined defocus levels. The system determined the defocus magnitude from a single measurement using the evaluation function. To enhance the validity of the results, the process was replicated three times. As illustrated in Figure 7a, the discrepancies in identification for various defocus amounts were all within a ±0.1 μm deviation, confined to a ±40 μm range. The system’s single-iteration operating range extends to ±40 μm, achieving an accuracy of 200 nm.

Figure 7b shows the results of continuous tests in which the objective was moved from negative to positive defocus points in 78 nm increments, with the output of the focusing system’s continuous-time sampling. During continuous-time sampling, the focal plane position of the output fluctuated within ±100 nm, which could be caused by environmental vibration or changes in laser intensity. This limited the focusing accuracy for taking a defocus photograph to 200 nm, which is also consistent with the results shown in Figure 7a. Nevertheless, the mean fluctuation tracked the shifts in defocus, as denoted by the green data in Figure 7b. The green data, which represents the averaged resampling outcomes, exhibited a closer alignment with the true defocus levels. This evidence suggests that the continuous measurement mode is capable of discerning defocus with 78 nm accuracy over a ±500 nm span.

The test results shown in Figure 7a indicate that the system maintains good focusing accuracy within a dynamic range of ±40 μm. Beyond ±40 μm, the accuracy decreases significantly. However, experiments have shown that within a defocusing range of ±100 μm, the maximum deviation does not exceed 10 μm, which is confirmed by the evaluation curve with R = 0.99216 in Figure 6b. Within the defocus range of ±100 μm, the defocus amount can be reduced to within 10 μm after the first iteration. The second iteration can utilize the defocus amount within a range of ±100 nm. There is a defocus amount within ±500 nm after two iterations; the third multisampling identification iteration shown in Figure 7b can achieve a focusing accuracy of 78 nm. Therefore, three iterations can achieve a focusing accuracy of 78 nm within a range of ±100 μm. In addition, increasing the number of iterations can further increase the dynamic range of the autofocus.

### 3.3. Sequencing Experiment

**Figure 8:**
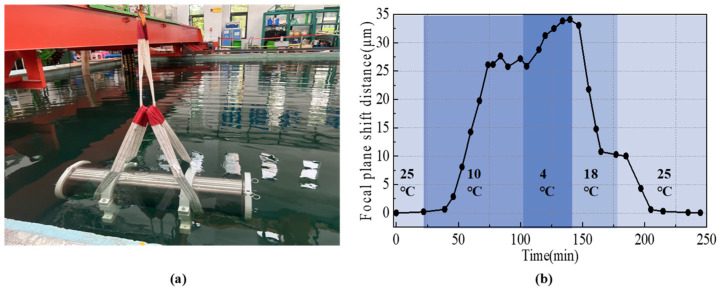
Temperature experiments in a simulated deep-sea environment. (**a**) Deep-sea gene sequencer. (**b**) Focal plane shift due to ambient temperature changes in the pressure-resistant cavity of a deep-sea, in situ gene sequencer.

The deep-sea sequencer is contained in a compressive cabin, as shown in Figure 8a, for long-term, in-situ deployment on the deep-sea floor. A series of deep-sea environment simulations were conducted to assess the impact of temperature fluctuations on the defocus levels of an imaging system within a pressure-resistant chamber for a prototype deep-sea gene sequencer. These simulations were conducted within a high and low-temperature environmental test cabinet (Cqhardy), which replicated conditions ranging from surface to deep-sea temperatures (4 °C to 25 °C).

As illustrated in Figure 8b, temperature fluctuations resulted in relative positional shifts between the object plane and the lens, with a maximum displacement of approximately 35 µm. Notably, there was a lag in the system’s response to environmental temperature changes that precluded effective compensation through environmental adjustments.

**Figure 9:**
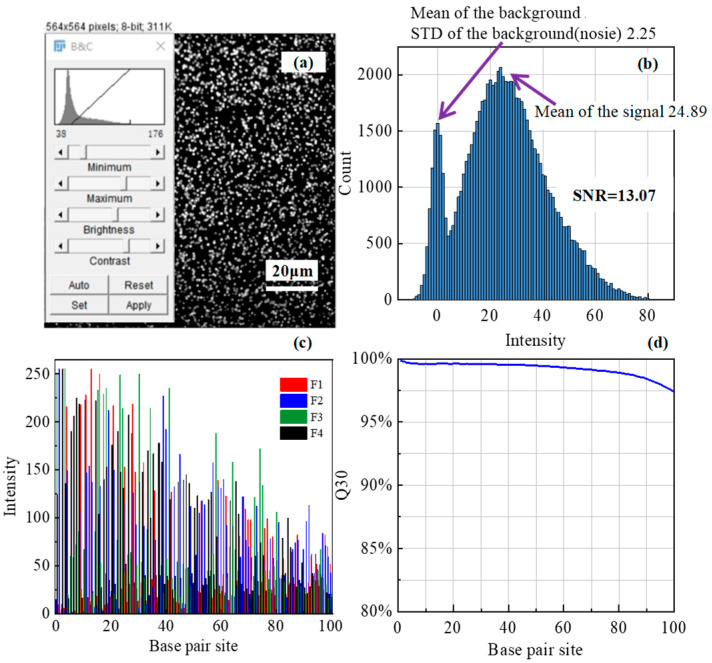
Standard nucleotide fragment sequencing experiment. (**a**) Grayscale histogram of local imaging on the chip. (**b**) Extracted intensity histogram. (**c**) Energy values of the four spectral channels with a single cluster. (**d**) Quality score Q30 values for overall data.

In our laboratory trials, we emulated the deep-sea’s low-temperature conditions and performed sequencing on an Illumina PhiX Control v3—using a prototype sequencer enabled with critical illumination autofocus—at 4 °C. The autofocused image of a chip segment, shown in Figure 9a, allowed for clear identification of base positions. The data was processed using background subtraction and Gaussian fitting, resulting in the intensity histogram shown in Figure 9b. Fluorescence information was characterized by higher grayscale values (average intensity of 24.89), while nonfluorescent background areas had lower grayscale values (average intensity of 0). The signal’s deviation above this background, which follows a normal distribution with a standard deviation (STD) of 2.25 for noise, led to an average signal-to-noise ratio of 13.07. This ratio was calculated by dividing the signal intensity by the noise intensity.

Fluorescence intensity data were extracted using a deep learning approach to obtain base sequences and associated quality metrics. Figure 9c shows the variation in fluorescence intensity at a given base position across four channels throughout the sequencing process. In gene sequencing, Q30 is used to assess the accuracy of sequencing data. Q30 indicates that approximately 1 in 1000 bases sequenced is likely to be incorrect and that sequencing results are 99.9% accurate. The Q30 value represents the proportion of DNA clusters positions identified at a 99.9% confidence level and is a key quality indicator in gene sequencing. Figure 9d illustrates the real-time Q30 quality scores. Defocusing significantly reduces image quality and thus significantly reduces the Q30 value. The sequencing process culminated in an impressive Q30 value of 97.36%, proving that accurate focus was maintained during sequencing.

## 4. Discussion

Testing has demonstrated that the deep-sea gene sequencer can sustain a maximum defocus of 35 μm within its operational temperature range. The device employs an autofocus method that relies on critical illumination, enabling it to achieve a focusing accuracy of 0.2 μm with a single capture and iteration over a defocus range of ±40 μm. This fulfills the stringent demands of deep-sea sequencing applications. The accuracy can be further refined by increasing the number of captures while adding iterations, which broadens the dynamic focus range. The detection limits of current systems are collectively determined by the objective lens’s magnification and focal length, the detector’s pixel size, and the width of the field of view.

Moreover, the applicability of this method extends beyond the in-situ use of gene sequencers in deep-sea environments. Researchers have the option to introduce separate wavelength laser illumination designed for focus detection, expanding its compatibility with existing gene sequencers and fluorescence microscopy systems. Additionally, the incorporation of an additional focus detection sensor would enable its integration as a standalone autofocus module for a variety of microscopy systems. Regardless of the shape of the laser illumination, an ideal focus detection signal edge should exhibit a “ridge-shaped” profile. The accuracy of the method is also contingent on the characteristics of the test surface; a smoother surface translates to enhanced accuracy. However, surface reflectivity is marginally relevant since the laser’s output power is significantly greater than that required for detecting focus. In addition, the edge detection algorithm, which is based on the distance between peaks and troughs of grayscale gradients, is an effective way of identifying the edges of defocused images.

## Data Availability

The original contributions presented in the study are included in the article/Supplementary Materials. Further inquiries can be directed to the corresponding authors.

## Supplementary Materials

The following supporting information can be downloaded at: https://www.mdpi.com/article/10.3390/s24155010/s1, gene sequence information of Illumina PhiX Control v3.
